# Charge Movements and Conformational Changes: Biophysical Properties and Physiology of Voltage-Dependent GPCRs

**DOI:** 10.3390/biom14121652

**Published:** 2024-12-23

**Authors:** Andreas Rinne, Moritz Bünemann

**Affiliations:** 1 Department of Biophysics and Cellular Biotechnology, “Carol Davila” University of Medicine and Pharmacy Bucharest, 050474 Bucharest, Romania; 2 Institute of Pharmacology and Clinical Pharmacy, Biochemical Pharmaceutical Center (BPC) Marburg, University of Marburg, 35043 Marburg, Germany

**Keywords:** GPCR, membrane potential, receptor signaling, voltage sensor

## Abstract

G protein-coupled receptors (GPCRs) regulate multiple cellular functions and represent important drug targets. More than 20 years ago, it was noted that GPCR activation (agonist binding) and signaling (G protein activation) are dependent on the membrane potential (V_M_). While it is now proven that many GPCRs display an intrinsic voltage dependence, the molecular mechanisms of how GPCRs sense depolarization of the plasma membrane are less well defined. This review summarizes the current knowledge of voltage-dependent signaling in GPCRs. We describe how voltage dependence was discovered in muscarinic receptors, present an overview of GPCRs that are regulated by voltage, and show how biophysical properties of GPCRs led to the discovery of voltage-sensing mechanisms in those receptors. Furthermore, we summarize physiological functions that have been shown to be regulated by voltage-dependent GPCR signaling of endogenous receptors in excitable tissues, such as the nervous system or the heart. Finally, we discuss challenges that remain in analyzing voltage-dependent signaling of GPCRs in vivo and present an outlook on experimental applications of the interesting concept of GPCR signaling.

## 1. Introduction

G protein-coupled receptors (GPCRs) belong to the class of seven transmembrane domain (7-TM) receptors, representing a large group of receptors embedded in the plasma membrane that control important cellular functions. There are more than eight hundred human GPCRs, which are ubiquitously expressed among tissues, and chronic receptor activation has been implicated in many diseases. Their pharmacological importance is underscored by the fact that more than 30% of all prescribed drugs target GPCRs [1,2]. Thus, there is a large interest in understanding how the direct environment of the receptor, such as signaling events at the plasma membrane, modulates GPCR activity. While it is now proven that many GPCRs are voltage-sensitive and that V_M_ regulates many physiological functions that involve endogenous GPCRs, a common molecular mechanism by which GPCRs sense voltage is still elusive. Voltage-sensitive proteins share two main properties: *(i)* they have an intrinsic mechanism to sense changes in membrane potential (V_M_) polarity (a voltage sensor) and (*ii*) changes in V_M_ induce a conformational change that affects signaling [3]. The following reviews our current knowledge of molecular mechanisms that render GPCRs voltage-sensitive.

## 2. Discovery of Gating Charge Movements and Conformational Changes in Response to Membrane Depolarization

More than 20 years ago, a paper by Ben-Chaim and coworkers demonstrated for the first time that the muscarinic M_2_ receptor (M_2_R) for acetylcholine (ACh) displayed an intrinsic voltage sensitivity that affected receptor signaling [4]. The M_2_R was less active at a positive membrane potential (+40 mV) than at a negative membrane potential (−60 mV), causing a right-ward shift of its concentration response to ACh. Because the readout for receptor activation was the M_2_R/ACh-induced K^+^ current (I_K,ACh_), caused by channel opening following binding of βγ-subunits released from G_i/o_ proteins but not by membrane depolarization itself [5], the authors concluded that the M_2_R protein must exhibit an intrinsic voltage sensitivity. This was later confirmed in a follow-up paper by the same group [6] that demonstrated the existence of gating charge movements within M_1_Rs and M_2_Rs in response to membrane depolarization, indicating conformational changes, which could be measured and quantified as currents analogous to the gating currents of voltage-gated ion channels. The authors also showed via ligand binding assays that the membrane potential modulated the receptor’s affinity for agonist binding. This was quickly recognized in the field of GPCR research and motivated other groups to study the novel properties of GPCRs [7]. Electrophysiological measurements of gating charge currents and the analysis of receptor signaling responses at various membrane potentials (voltage/response curves) allowed researchers to calculate the quantity and movement of elementary charges during voltage-induced conformational changes. Compared with the charges that move during activation of proteins containing well-characterized voltage sensors, such as helix S4 in voltage-gated ion channels (e.g., >4 for the canonical Shaker-K^+^ channel) [3], the reported z-values in GPCRs are smaller and ≤1 (Table 1). This difference in elementary charges already indicates that a potential voltage sensing mechanism in GPCRs may differ from molecular gating mechanisms of other voltage-sensitive proteins. Multiple studies have provided values of V_M_ at which GPCRs showed half-maximal activation (V_50%_, Table 1). So far, all reported values lie in the range of physiological membrane potential. Consequently, in electrically excitable tissues, the signaling of endogenous GPCRs depends on V_M_, as discussed below.

## 3. On the Search for a Voltage Sensor: Does an Internal Na^+^ Ion Play a Role?

In principle, charged residues of amino acids or dipoles within proteins can move when the electrical field across the membrane changes its polarity and serve as potential voltage sensors [3]. The small number of moving elementary charges in GPCRs may indicate that charged amino acid residues, ions, or dipoles may serve as voltage sensors. Activation of receptors by agonists depends on extracellular Na^+^; the identification of a conserved, internal Na^+^ ion binding site in crystal structures of class A GPCRs has drawn attention to the Na^+^ ion as a putative voltage sensor. Binding of this internal “allosteric Na^+^” stabilizes the inactive receptor conformation. Na^+^ ions enter the receptor core from the extracellular side and Na^+^ binding is coordinated by a conserved aspartate residue, D^2.50^ of TM2 [24,25]. Following agonist binding and a change of the GPCR into its active configuration, the Na^+^ binding pocket collapses and the Na^+^ ion is probably released to the cytoplasm [26]. Thus, during each receptor activation cycle (from inactive conformation to agonist-bound conformation and back), one Na^+^ ion is moved from the extracellular space to the cytoplasm, indicating that the receptor–Na^+^ interaction is dynamic. The conserved Na^+^ ion was quickly hypothesized to be a bona fide voltage sensor common to all class A GPCRs [27,28]. This assumption was supported by molecular dynamics (MD) simulations: Vickery and coworkers analyzed the relationship between internal Na^+^ binding and voltage dependence. In silico simulations revealed that Na^+^ binding was dynamic, and membrane depolarization caused a dislocation of the Na^+^ ion from its internal binding site. Interestingly, this movement could account for charge movements matching the previously measured gating charge currents in M_2_Rs and opioid receptors [27]. On the other hand, when the Na^+^ binding site was mutated in receptors in vitro, it became clear that some receptors did not rely solely on the Na^+^ ion mechanism to exhibit their voltage dependence. Substitutions of D^2.50^ with non-charged amino acids rendered M_2_Rs less sensitive to ACh, but did not remove their voltage dependence [29,30]. Likewise, omitting extracellular Na^+^ or preventing internal Na^+^ binding by D^2.50^ charge neutralization did not abolish the voltage sensitivity of 5-HT_1A_ receptors [8]. The experimental analysis of D^2.50^ receptor mutants can be challenging because some mutants display strongly reduced G-protein signaling efficacies, which renders functional readouts and the interpretation of receptor signaling and pharmacology difficult [26,30]. While the studies presented above can be interpreted in favor or in disapproval of the Na^+^ mechanism, there is another important caveat; it is simply unknown whether D^2.50^ receptor mutants do not bind Na^+^ at all or whether Na^+^ binding is coordinated by alternative residues that contribute to the Na^+^ binding site, for example, S^3.39^ [24].

**Figure 1:**
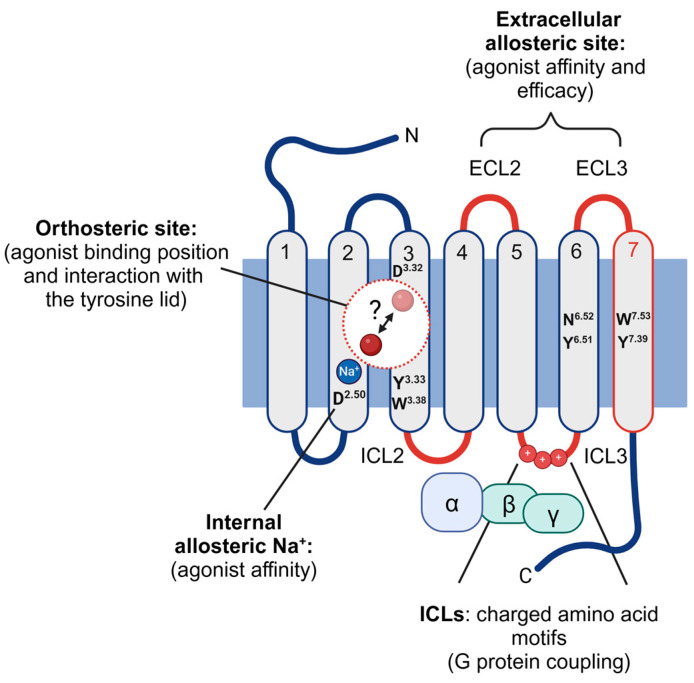
Receptor regions involved in the voltage sensitivity of muscarinic GPCRs. Graphical summary of amino acids (black) and receptor regions (red) that have been implicated in voltage-sensing mechanisms in muscarinic receptors. Amino acid residues are numbered according to the numbering scheme of Ballesteros and Weinstein [33]. D^2.50^ coordinates Na^+^ binding. D^3.32^ affects agonist binding position. N^6.52^ affects agonist binding position. Y^3.33^, Y^6.51^, Y^7.39^ form the tyrosine lid and affect agonist binding and G-protein coupling. W^3.28^, W^7.53^ contribute to allosteric modulation of receptor function. ECL: extracellular loop. ICL: intracellular loop. For further details and references please see the text. Created in BioRender. Rinne, A. (2024) BioRender.com/i41w075 (accessed on 19 December 2024).

There is, however, other evidence that a voltage-sensing mechanism in GPCRs could be more complex: Navarro-Polanco and coworkers screened the sequence of the M_2_R for single amino acids that were either charged and exposed to the electrical field across the membrane (e.g., D^3.32^) or critically involved in agonist binding (W^3.28^, Y^3.33^, Y^6.51^) and addressed the functional impact of those residues on the development of gating charge movements through point mutations [19]. As an outcome, the individual point mutations altered the pharmacological properties of the mutant receptors; but did not abolish their voltage sensitivity. The authors concluded that those residues were instead involved in an allosteric pathway that couples a voltage sensor to the receptor function, while not serving as voltage sensors per se. This agrees with calculations to determine the energy required to alter the activation state of GPCRs in response to changes in membrane potential polarity [31]. Zhang and coworkers concluded from their calculations that a voltage-sensing mechanism may involve multiple charged residues or dipoles that are distributed across the entire ligand–receptor complex, rather than a single charged amino acid. Thus, other receptor regions may contribute to the phenomenon of voltage sensitivity as well, as described below. A comprehensive list of single amino acids implicated in the voltage dependence of GPCRs is given in reference [32]. Figure 1 summarizes receptor regions that have been implicated in voltage sensing, described here and below.

## 4. The Impact of the Ligand Structure on the Voltage Dependence of GPCRs

Agonists bind either to GPCRs close to the extracellular receptor surface or to a binding pocket that sits deep in the receptor core within the plasma membrane [34,35,36]. Both receptor regions are exposed to changes in electrical field polarity [31]. Several studies reported that the affinity of the receptor for the agonist was controlled by V_M_, translating into more efficient or less efficient receptor signaling during membrane depolarization [4,9,10,20]. Those observations brought into focus the orthosteric binding site for agonists. Regarding muscarinic receptors, it was observed that an outcome of voltage-sensitivity activation or deactivation was dependent on the type of agonist that was used to activate the receptor. For example, ACh-activated M_2_Rs and M_3_Rs displayed deactivation in response to membrane depolarization, which turned into activation when the receptors were simply activated with the structurally different agonist pilocarpine [17,19,37]. MD simulations were used to analyze the molecular interactions of ligands with residues of the orthosteric binding pocket in M_3_Rs, showing that the full agonists ACh and carbachol (CCh) adopted similar binding positions within the orthosteric binding site that were notably different from the one observed for the partial agonist pilocarpine. Each individual agonist’s binding position could be correlated with positive or negative modulation of the M_3_R by V_M_ [17]. Similar agonist dependencies were described by Kirchhofer and colleagues for opioid receptors; it was shown that membrane depolarization caused either activation (for morphine) or deactivation (for fentanyl), an effect that was also attributed to specific subsets of agonist binding poses in MD simulations [38]. Importantly, both of these studies showed that point mutations within the orthosteric site that altered agonist binding positions in MD simulations also changed the direction of voltage dependence in experiments with live cells [17,38]. A ligand-structure-dependent voltage sensitivity was also reported in studies by Salholm and coworkers, who showed that the extent of voltage dependence in D_2_S dopamine receptors (deactivation by depolarization) was largest for receptors that were activated by dopamine but was reduced when synthetic analogs were used [13,39]. In addition, Hazan and coworkers demonstrated recently that the binding of the antagonists atropine and scopolamine to M_2_Rs was dependent on V_M_, suggesting that the inactive ligand–receptor complex was also voltage-dependent [40]. Among the very complex agonist–receptor interactions that were specific for each ligand and receptor subtype, it was noted that binding of some agonists involved residues of TM3 or TM6 [17,38]. Both helices are relevant for receptor activation; for example, some TM3-TM6 interactions stabilize the inactive GPCR conformation and set agonist affinity [35,36], while movement of TM6 is characteristic of the transition from the inactive to the active receptor state and is required for G-protein coupling [41,42,43].

An open question for future studies is whether charged ligand molecules move freely within their binding pocket in response to a change in the electrical field polarity, or whether they adopt different ligand binding poses in response to a conformational change of the receptor protein following membrane depolarization. However, the observation that class C mGluRs, in which the ligand binds to large extracellular domains that are not exposed to the electrical field across the plasma membrane, are voltage-sensitive [20] implies that the modulation of agonist binding is not the only mechanism that affects voltage sensitivity. Alternative receptor regions that contribute to the voltage sensitivity in GPCRs are discussed next.

## 5. G-Protein Coupling, Intracellular Receptor Loops, and the Voltage Dependence of GPCRs

Ben-Chaim and colleagues observed that ACh-activated M_1_Rs, which couple to G_q_ proteins, were positively modulated by voltage, but ACh-activated M_2_Rs, which couple to G_i/o_ proteins, were deactivated by voltage [6]. This led to the hypothesis that G-protein coupling or the type of G protein may somehow affect the outcome of voltage dependence in GPCRs. We know now that the physical receptor–G-protein interaction itself is not required for voltage sensitivity in GPCRs. For example, the alpha_2A_ adrenergic receptor (α_2A_AR) and the M_1_R remained voltage-sensitive after receptors and G proteins were functionally uncoupled with pertussis toxin (PTX) or GTPγS, respectively [9,17]. Furthermore, M_1_Rs and M_3_Rs, both of which couple to G_q_, displayed voltage dependencies with opposite polarities, arguing against the idea that the type of G protein was a factor [17]. Indeed, Table 1 shows that the outcome of membrane depolarization (activation or deactivation) is not correlated with a specific class of G protein. However, there is experimental evidence that intracellular loops (ICLs), which are involved in G-protein coupling, affect voltage dependence in GPCRs; a sequence comparison between ICL3 of M_1_R and M_2_R identified a charged, basic N-terminal motif (KDKK) within ICL3 of M_2_R that was not present in ICL3 of M_1_R. The latter carries the uncharged motif ELAAL at this location. When the M_2_R motif was replaced with ELAAL, the mutant M_2_R was arrested in a voltage-insensitive, high-affinity state for agonists [6], an effect that could be confirmed by exchanging a similar motif in the *Drosophila* muscarinic receptor MR-A [44]. Ohana and coworkers confirmed that ICLs influenced the voltage sensitivity of mGluRs, in a study that analyzed the voltage dependencies of mGluR_1_ and mGluR_3_ [20]. A sequence comparison of ICL1, ICL2, and ICL3 between the two receptor subtypes revealed that the distribution pattern of charged residues within those loops differed. Chimeric receptors based on mGluR_1_ (activated by depolarization) that carried either ICL1 and ICL2 or ICL2 and ICL3 of mGluR_3_, adopted the voltage-dependent phenotype of mGluR_3_ (deactivated by depolarization). Using radioactive ligand-binding experiments, the authors showed that V_M_ modulated the affinity of those receptors for glutamate binding, which in turn was affected by the amino acid sequences of ICL2 and ICL3 [20]. Another example is the voltage sensitivity of dopamine receptors; D_2S_R is voltage-dependent (deactivated by depolarization), whereas the closely related D_3_R is voltage-insensitive. A sequence analysis identified an alternative motif within ICL3 (KQQSLKRRSMT) where D_2S_R and D_3_R sequences differed the most. Interestingly, a chimera of D_2S_R carrying the corresponding ICL3 motif stemming from D_3_R (RLPVGRPQLPGL) adopted the voltage-insensitive phenotype of D_3_R [45]. It is not clear whether these charged motifs directly serve as voltage sensors or whether they stabilize a certain receptor conformation in which a voltage sensor is coupled to the agonist binding site, as suggested by Ben-Chaim and colleagues [6]. Because the ICL/G-protein/receptor interaction controls the affinity of GPCRs for their ligands [46], those experiments confirm the central role of V_M_ in controlling agonist binding and thus, the signaling efficacy of GPCRs. Alternatively, extracellular receptor regions can control affinity for agonists. Their contribution to the voltage dependence of GPCRs is discussed next.

## 6. Extracellular Receptor Loops, Allosteric Modulation, and the Voltage Dependence of GPCRs

Allosteric modulation of GPCRs refers to the binding of small molecules to receptor regions that are distinct from the orthosteric site and which influence receptor signaling [47]. So-called allosteric modulators (AMs) cause either further activation (positive AMs) or attenuation of receptor signaling (negative AMs) [48]. Allosteric modulation of GPCRs has been widely studied in MRs, due to the availability of many commercially available allosteric compounds. Analysis of crystal structures bound to AMs helped to identify allosteric sites and reveal how allosteric modulators interact with the orthosteric site in GPCRs [49]. MRs comprise five members (M_1_Rs to M_5_Rs), which are highly conserved at the receptor regions that constitute the orthosteric binding site but differ at their extracellular allosteric binding site, which is composed of residues stemming from extracellular loops (ECLs) 2 and 3 and residues of TM7 [50,51]. Because the modulation of GPCR signaling by V_M_ resembles some properties of allosteric modulation, we and others analyzed whether the extracellular allosteric site was involved in the voltage sensitivity of MRs. Hoppe and coworkers reported that allosteric chimeras of muscarinic receptors in which the M_1_R (activated by depolarization for ACh) carried ECL2, ECL3, and TM7 of M_3_R (inactivated by depolarization for ACh) and vice versa, displayed either strongly reduced voltage dependence or resulted in voltage-insensitive receptors, respectively [18]. An alternative approach showed that the single point mutation W^7.35^A, which was reported to impair the interaction of the extracellular allosteric site with the orthosteric agonist binding site in M_2_Rs [52], abolished the voltage dependence of the M_3_R entirely [18]. In this context, it is worth noting that AMs bound to the extracellular allosteric sites of M_1_R [18] or M_2_R [40] did not affect voltage dependencies in the presence of ACh. This suggests that classical AMs modulate agonist binding via an intramolecular network that is at least partially different from the one that mediates modulation of agonist binding by V_M_. Nevertheless, both mechanisms share the feature that they interact with the orthosteric agonist.

Another important structural element that mediates interaction of allosteric and orthosteric sites specific to muscarinic receptors is the so-called “tyrosine lid,” consisting of three conserved tyrosine residues, Y^3.33^ of TM3, Y^6.51^ of TM6, and Y^7.39^ of TM7. These three residues are loosely related to each other in inactive receptors but assemble and close the orthosteric site like a lid once an agonist is bound. The tyrosine lid represents an important interface at which the extracellular allosteric site interacts with the agonist. It has been implicated in controlling agonist affinity [50] as well as receptor signaling efficacy (G-protein binding) [53]. Barchad-Avitzur and colleagues identified the tyrosine lid as a pivotal structure that could serve as a voltage sensor in M_2_Rs [30]. They proposed a mechanism through which the hydroxyl groups of the tyrosine residues create dipoles that can move within the electrical field. The authors demonstrated that tyrosine-to-phenylalanine mutations removed depolarization-induced changes in agonist affinity, and thus, voltage dependence. They suggested a molecular mechanism through which hyperpolarizing membrane potential stabilizes the lid (and agonist binding) and depolarization-induced movement of tyrosine residues destabilizes the lid, causing low-affinity conformation of the M_2_R for agonists [30]. While this mechanism held true for M_2_Rs, corresponding tyrosine mutations of all three residues in M_3_Rs markedly reduced the affinity of the mutant receptor for ACh but did not abolish its voltage dependence entirely [18]. This is in line with a study showing that the tyrosine lid modifies agonist affinity and signaling efficacy in different ways in M_2_Rs vs. M_3_Rs [54]. Hoppe et al. showed in MD simulations that the allosteric residue W^7.35^ stabilizes this lid; they proposed a molecular mechanism through which the mutation W^7.35^A may disrupt allosteric modulation in MRs by chemical modulators or by membrane depolarization via destabilization of the tyrosine lid [18]. The tyrosine-lid sensor mechanism is compatible with the small number of elementary charges that move during depolarization. Like the allosteric internal Na^+^ ion, the tyrosine lid sets the affinity for agonist binding, which in turn is modulated by V_M_.

## 7. Physiology of Voltage-Dependent GPCRs

Voltage regulates GPCR activity in the range of physiological values (Table 1), which leads to the question of whether the physiological functions of endogenous receptors are voltage-sensitive in vivo. Even before the concept of voltage-sensitive GPCR signaling was established, there was experimental evidence that receptor-mediated Ca^2+^ release from intracellular Ca^2+^ stores, a process not involving voltage-gated Ca^2+^ channels, was voltage-dependent. An early study by Marty and coworkers demonstrated that ACh-induced liberation of Ca^2+^ from the endoplasmic reticulum in rat lacrimal acinar cells was dependent on V_M_ [55]. Likewise, Ong and coworkers showed that membrane hyperpolarization increased but membrane depolarization decreased the spike frequency of ACh-induced Ca^2+^ oscillations in mouse pancreatic acinar cells, in which Ca^2+^ oscillations are known to control vesicular secretion rates [56]. This effect was dependent on the concentration of ACh, suggesting that the voltage dependence of Ca^2+^ signaling was a function of muscarinic receptors. More recently, a series of papers by Martinez-Pinna and colleagues showed that the activation of metabotropic P2YRs by extracellular ADP was potentiated at positive membrane potentials in rat megakaryocytes, which are precursor cells of thrombocytes [23,57]. This sensitized the liberation of Ca^2+^ from intracellular stores, which is an important cellular signal for thrombocyte activation [58]. Gurung and coworkers also demonstrated that the modulation of P2YRs by V_M_ was well pronounced when a low, physiological concentration of 30 nM ADP was used to activate the receptor [59]. The studies above clearly demonstrate that endogenous Ca^2+^ signals are controlled by the voltage dependence of GPCR signaling. This has physiological relevance, because non-excitable cells can experience membrane depolarizations of more than 60 mV that control important cellular functions such as cell cycle progression [60]. These changes in V_M_ are sufficient to modulate GPCRs.

**Figure 2:**
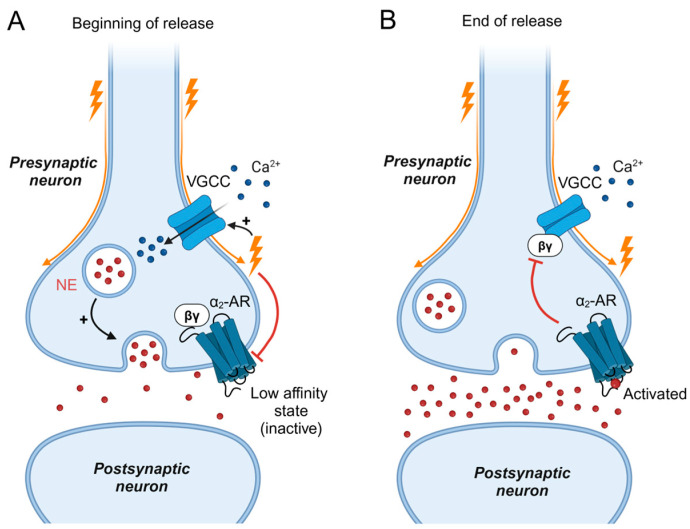
Proposed role of voltage-dependent α_2A_AR signaling in regulating neurotransmitter release. In presynaptic neurons, the α_2A_AR terminates release of norepinephrine (NE) by activation of G_i/o_ proteins. The released βγ-subunit directly inhibits Ca^2+^ influx via N-type VGCCs, preventing vesicle fusion and neurotransmitter release [71]. The receptor’s voltage sensitivity fine- tunes this mechanism. (**A**) At the beginning of release, membrane depolarization reduces the affinity of the receptor for NE and the inhibitory feedback mechanism is inactive when the concentration of NE is low within the synaptic cleft. This type of receptor inhibition prevents premature termination of release. (**B**) When release progresses and the concentration of NE increases, the voltage-sensitive component of the receptor is ineffective and robust receptor activation terminates release. Created in BioRender. Rinne, A. (2024) BioRender.com/i41w075 (accessed on 9 November 2024).

Neuronal GPCRs are either part of a negative feedback mechanism to control the timing of neurotransmitter release (presynaptic receptors, Figure 2) or they control postsynaptic cellular functions [61]. A study that analyzed the kinetics of voltage-induced charge movements in M_2_Rs concluded that on- and off-currents, reflecting conformational switches of the receptor molecule following the onset and offset of a membrane-depolarizing pulse, are fast enough (~0.3 ms for the on response) to occur during brief ms-long depolarizations of the neuronal action potential [62], suggesting that neuronal action potential can modulate GPCRs. The same group demonstrated that the voltage-dependent component of M_2_R signaling adds another layer to the control of neurotransmitter release, in addition to the well-known Ca^2+^-dependent release mechanism, the “Ca^2+^-hypothesis” of neurotransmitter release [63]. By measuring quantal ACh release at the mouse diaphragm neuromuscular junction, they demonstrated that the voltage dependence of the presynaptic M_2_R set the right affinity of the receptor for its negative feedback loop. This was directly demonstrated in M_2_R knockout mice, in which isolated, Ca^2+^-dependent neurotransmitter release failed to reproduce the precise timing of release in wild-type animals, causing prolonged neurotransmitter release [64]. This novel mechanism has been coined “Ca^2+^-voltage hypothesis” of neurotransmitter release, by its authors [65]. Another type of regulation of neurotransmitter release by voltage sensitive GPCRs has been detected for P2YRs, whose voltage dependence (activation by depolarization) facilitated catecholamine release in sympathetic chromaffin cells [66]. Thus, voltage dependence fine-tunes the affinity of the presynaptic feedback receptors during neurotransmitter release. The voltage dependence of presynaptic α_2A_ARs, which negatively control norepinephrine (NE) release [67], could also resolve a pharmacological puzzle; EC_50_ values of native and recombinant α_2A_AR have been reported in the lower nanomolar range (~10 nM) [68,69], whereas NE concentrations measured in synaptic clefts during neurotransmitter release quickly reached concentrations in the µM range (≥10 µM), depending on tissue type and synaptic cleft geometry [70]. So how can a very sensitive adrenergic receptor control NE release without ending it prematurely? Despite spatial separation of transmitter release zones and feedback receptor locations, the answer may lie partially in its voltage dependence. The α_2A_AR was deactivated by membrane depolarization, an effect that was well pronounced at low to moderate concentrations of NE, but its voltage dependence was abolished when the receptor was activated by saturating, micromolar concentrations of NE [9]. Thus, it is plausible that at the very beginning of release, membrane depolarization sets the receptor to a state of low affinity, allowing release to happen (Figure 2A). At the end of release, higher NE concentrations “override” the receptor’s voltage dependence and the feedback loop terminates neurotransmitter release (Figure 2B).

If neurotransmitter release is affected by the voltage dependence of GPCRs, then one can assume that systemic functions of the brain might be controlled by the voltage sensitivity of GPCRs. A recent paper by Rozenfeld and coworkers demonstrated a physiological role for the voltage dependence of a muscarinic *Drosophila* receptor (MR-A) in vivo [44]. The authors found that membrane depolarization potentiated the activity of MR-A, analog to the mammalian M_1_R [6,17,18]. By generating a fruit fly that expressed a voltage-insensitive mutant of the MR-A receptor, they demonstrated that voltage-dependent signaling of MR-A was implicated in the neuronal plasticity, learning, memory, and odor-response behavior of the fruit flies. Taken together, the voltage sensitivity of endogenous neuronal receptors contributes to the fine tuning of physiological functions of the brain, such as neurotransmitter release and systemic organ functions.

Another important electrically excitable organ is the mammalian heart, where muscarinic receptors mediate the responses to ACh released by the vagus nerve. One main effector downstream of cardiac M_2_Rs is the ACh-activated K^+^ current (I_K,ACh_), mediated by G-protein-activated, inwardly rectifying K^+^ (GIRK) channels [72]. A series of papers by Moreno-Galindo and colleagues demonstrated that one important function of the voltage control of M_2_R activity lies in shaping the ion channel’s response to ACh. They showed that the degree of the channel’s reduction in amplitude during prolonged activation, a well-known channel property that they termed “gating relaxation,” by those authors or “acute desensitization” by others, could be attributed to the changes in affinity that the M_2_R experienced during changes in V_M_. Remarkably, the observed relaxations in I_K,ACh_ current reflected the opposite directions of M_2_R voltage dependence when activated with pilocarpine instead of ACh [73]. Because membrane depolarization reduced the affinity of endogenous M_2_Rs for ACh, I_K,ACh_ in atrial myocytes deactivated approximately twice as fast at +50 mV than at −100 mV, suggesting that the same ACh stimulus could evoke different channel responses depending on V_M_ [37]. Taken together, these papers demonstrated that the voltage-dependent component of cardiac M_2_R signaling shapes the amplitude and kinetics of a physiological effector at the endogenous expression levels of all components of this signaling cascade. Therefore, the intrinsic voltage sensitivity of cardiac M_2_Rs contributes to the fine-tuning of GIRK channel activity, which in turn controls important cardiac repolarization events, such as slowing down the frequency of sinoatrial pacemaker cells [72].

When studying electrically excitable tissues or primary cell preparations, it may be difficult to discriminate the contribution of voltage-sensitive GPCRs to a cellular function in the surroundings of other active voltage-sensitive proteins such as voltage-gated ion channels. This has prompted some authors to question the entire concept of voltage dependence in muscarinic receptors, suggesting that voltage sensitive signaling of muscarinic receptors could rather be explained by co-activation of voltage-gated Na^+^ channels in certain tissue preparations such as synaptosomes [74]. The author concluded that membrane depolarization is sensed by the ion channel, which interacts with the M_2_R protein directly. The conformational change of the channel during membrane depolarization is then transferred to the receptor and results in an increase in agonist affinity and G_o_ -protein activation without any contribution of a voltage sensor embedded in the receptor. We are not sure whether one can extrapolate this principle to other cell types or tissue preparations. First, membrane depolarization did not enhance but reduced the signaling strength of endogenous M_2_Rs in cardiac myocytes in the presence of endogenous Na^+^ channels [73]. Second, conformational changes were observed in M_2_Rs (gating charges) and other GPCRs (optical biosensors) when analyzed in heterologous expression systems in the absence of voltage-gated Na^+^ channels [9,10,22]. This was the case for most of the studies cited in Section 2, Section 3, Section 4, Section 5 and Section 6 of this review, suggesting that there is indeed an intrinsic mechanism for voltage sensitivity in GPCRs [75].

## 8. Conclusions and Outlook

The studies presented above clearly show that GPCR signaling is voltage-dependent, which is a signaling property relevant for physiological signaling. What is the nature of a voltage sensor in GPCRs? The different mechanisms presented above can be interpreted in the following ways. The first interpretation is that some receptors have developed individual mechanisms to sense voltage. The second interpretation is that there is one conserved mechanism to sense voltage, but the molecular pathways through which a putative voltage sensor is coupled to induce conformational changes and receptor signaling may differ between individual receptor types. The first scenario would imply that there are several activating mechanisms with different biophysical properties. In the second scenario, the biophysical properties for sensing membrane depolarization would be similar for all receptor types. The biophysical data reported so far (Table 1) show that z-values and V_50%_ values are indeed similar for different receptor types, which would support the second interpretation. A third and completely alternative mechanism for voltage sensing has been suggested by Zhang and coworkers. Based on simulations, they propose that the overall charge distribution of the receptor can be modified by depolarization-induced changes in the plasma membrane properties at receptor–lipid interfaces, which would result in conformational changes of the receptors [31]. They conclude that this could affect GPCR activity in response to membrane depolarization, without the requirement for a voltage sensor encoded within the receptor protein. So far, such a contribution of changes in the lipid bilayer to the phenomenon of voltage sensitivity in GPCRs has not been experimentally addressed. Thus, the molecular identification of the voltage sensor in GPCRs is still an important and interesting goal for future studies. Another future direction for this type of signaling research will be the description of more physiological processes that are regulated by voltage-dependent GPCRs in vivo. As discussed above in the context of receptor mutants, one drawback of such studies may be reflected in the fact that voltage-insensitive receptor mutants often displayed weak signaling properties that were due to low affinities for agonists and insufficient efficacy to activate G proteins. This raises the question whether those mutants would signal properly at low, physiological concentrations of agonists in tissues. The implementation of novel computational methods that can predict the functional consequences of mutations in relation to proteins will probably aid in generating more voltage-insensitive GPCR mutants that preserve their native pharmacological and signaling properties [76]. Such mutants could then improve future studies to further delineate the role of voltage-dependent GPCR signaling in vivo.

## Figures and Tables

**Table 1:** Biophysical properties of voltage-sensitive GPCRs.

Receptor	G protein	Depolarization	z-Values	V_50%_	Agonist	Reference
5HT_1A_R	G_i/o_	deactivates	n.d.	n.d.	5-HT Tandosp.	[8]
α_2A_AR	G_i/o_	deactivates	0.5	−20 mV	NE	[9]
β_1_AR	G_s_	deactivates	0.49 0.36	−27 mV −28 mV	Adr Iso	[10]
CB_1_R	G_i/o_	activates	0.8	−45 mV	2-AG	[11]
D_1_R	G_s_	deactivates	n.d.	n.d.	DA	[12]
D_2S_R	G_i/o_	deactivates	n.d.	n.d.	DA	[13]
D_5_R	G_s_	deactivates	n.d.	n.d.	DA	[12]
EP_3_R	G_s_	deactivates	n.d.	n.d.	Iloprost	[14]
FPR	G_q_	activates	n.d.	n.d.	U46619	[14]
H_3_R	G_i/o_	deactivates	n.d.	n.d.	His	[15]
H_4_R	G_i/o_	deactivates	n.d.	n.d.	His	[15]
LPAR	G_q_	activates	n.d.	n.d.	LPA	[16]
M_1_R	G_q_	activates	0.76 1.07	−53 mV −51 mV	CCh Ach	[17] [18]
M_2_R	G_i/o_	deactivates	0.55 0.85	−44 mV −55 mV	Ach	[6] [19]
M_3_R	G_q_	deactivates	0.65	−42 mV	Ach	[18]
mGluR_1a_	G_q_	deactivates	n.d.	n.d.	Glu	[20]
mGluR_3_	G_i/o_	deactivates	n.d.	n.d.	Glu	[20]
mGluR_5_	G_q_	deactivates	n.d.	n.d.	Quis	[21]
µ-Opioid-R	G_i/o_	activates	0.66	−6 mV	Mor	[22]
P2Y_1_R	G_q_	activates	n.d.	n.d.	ADP	[23]
TPR	G_q_	activates	0.5	−46 mV	U46619	[14]

Abbreviations (receptors): 5HTR: 5-hydroxytryptamine receptor; αAR: alpha adrenergic receptor; βAR: beta adrenergic receptor; CBR: cannabinoid receptor; DR: dopamine receptor; EPR: prostaglandin E receptor; FPR: prostaglandin F receptor; HR: histamine receptor; LPAR: lysophosphatidic acid receptor; mGluR: metabotropic glutamate receptor; P2YR: purinergic receptor type 2Y; TPR: thromboxane receptor. Lowercase letters and numbers indicate receptor subtypes. Abbreviations (agonists): 2-AG: 2-arachidonoylglycerol; 5-HT: 5-hydroxytryptamine; ADP: adenosine diphosphate; Adr: adrenaline; CCh: carbachol; DA: dopamine; Glu: glutamate; His: histamine; Iso: isoprenaline; LPA: lysophosphatidic acid; Mor: morphine; NE: norepinephrine; Quis: quisqualate; Tandosp.: tandospirone. Other: n.d.: not determined. G proteins: receptor G-protein preference refers to the type of G protein reported in the corresponding reference. Lowercase letters indicate G-protein classifications based on the alpha subunit of heterotrimeric G proteins [18].

## Data Availability

No new data were created or analyzed in this study. Data sharing is not applicable to this article.
